# Tailoring particle translocation via dielectrophoresis in pore channels

**DOI:** 10.1038/srep31670

**Published:** 2016-08-16

**Authors:** Shoji Tanaka, Makusu Tsutsui, Hu Theodore, He Yuhui, Akihide Arima, Tetsuro Tsuji, Kentaro Doi, Satoyuki Kawano, Masateru Taniguchi, Tomoji Kawai

**Affiliations:** 1 The Institute of Scientific and Industrial Research, Osaka University, Ibaraki, Osaka 567-0047, Japan; 2 Department of Mechanical Science and Bioengineering, Graduate School of Engineering Science, Osaka University, Toyonaka, Osaka 560-8531, Japan; 3 School of Optical and Electronic Information, Huazhong University of Science and Technology, Luo Yu Road, Wuhan 430074, China

## Abstract

Understanding and controlling electrophoretic motions of nanoscopic objects in fluidic channels are a central challenge in developing nanopore technology for molecular analyses. Although progress has been made in slowing the translocation velocity to meet the requirement for electrical detections of analytes via picoampere current measurements, there exists no method useful for regulating particle flows in the transverse directions. Here, we report the use of dielectrophoresis to manipulate the single-particle passage through a solid-state pore. We created a trap field by applying AC voltage between electrodes embedded in a low-aspect-ratio micropore. We demonstrated a traffic control of particles to go through center or near side surface via the voltage frequency. We also found enhanced capture efficiency along with faster escaping speed of particles by virtue of the AC-mediated electroosmosis. This method is compatible with nanopore sensing and would be widely applied for reducing off-axis effects to achieve single-molecule identification.

Solid-state nanopores are a powerful bioanalytical tool capable of counting and discriminating individual nanoscopic objects in liquid^1^,^2^,^3^ that promises applications in biosensing from single-molecule sequencing^4^,^5^,^6^,^7^,^8^ to biomolecule screening^9^,^10^. In this sensor, analytes are electrophoretically driven to pass through a hole in a membrane by imposing an electric voltage between *cis* and *trans* compartments, which causes temporal blocking of ionic current in the fluidic channel upon the single-particle trafficking^3^. As larger substances can exclude more ions, the resistive ionic signatures enable identification of individual particles by their size^11^.

In nanopore sensing, rapid translocation motions of analytes have been a common issue that poses a technical difficulty to detect a molecular signature with enough spatial resolution as it necessitates a high-gain current amplifier with incredibly wide bandwidth^12^,^13^,^14^. Much effort has thus been devoted to incorporate additional force field in a fluidic channel to achieve better control of the electrokinetics involved in the resistive pulse sensing, such as optical/electrical gating^15^,^16^,^17^, optical tweezer^18^, and salt gradient^19^, which led to significant improvement in slowing down the longitudinal electrophoretic velocity. In contrast, little work has been done on the particle motions in transverse directions, albeit the fact that fluctuations in the radial positions effectively affect the electrical signals^20^,^21^,^22^ thereby leading to serious degradation of the sensor accuracy.

We herein show that dielectrophoretic traps can be used to restrict the motional degrees of freedom of particles in the radial directions inside a pore whereby enabling regulation of the translocation dynamics. Dielectrophoresis is a manipulation technique that implements alternative electric fields to induce dipoles on particles such as cells and proteins so as to move them along the potential gradient through Coulomb interactions^23^,^24^,^25^. Therefore, unlike the conventional DC methods in gating nanopores wherein electrostatic cross-link between the multiple sources and accompanied unpredictable influence on the electrical signatures is inevitable^15^,^26^,^27^, dielectrophoresis is an AC approach whose effects can be separated from the DC components by low-pass filtering.

Our device consists of four Pt nanoelectrodes embedded in a micropore sculpted in a Si_3_N_4_ membrane (Figs 1a–c and S1). The pore channels were designed to have a low thickness-to-diameter aspect-ratio structure (thickness 100 nm, diameter 1.4 μm) so as to exploit the expansive electrical sensing zone to evaluate the field effects on the capture-to-translocation processes^22^. The open pore conductance *G* of 103 nS was deduced by measuring the ionic current *I*_ion_ flowing through the pore using two Ag/AgCl electrodes while increasing the electrophoretic voltage *V*_b_ (Fig. 1d) in TE buffer (pH 8.0, Tris-HCl 10 mM, EDTA 1 mM), which agrees with *G* = 1/(*R*_acc_ + *R*_pore_) with the effective pore diameter *d*_pore_ = 1.3 μm and thickness 80 nm, where *ρ* = 12 Ωm, *R*_acc_ = *ρ*/*d*_pore_ = 9.2 MΩ, and *R*_pore_ = *ρπd*_pore_^2^/4*L*_pore_ = 0.72 MΩ are the resistivity of the ionic solution, the access resistance (i.e. the ionic resistance from the electrodes to the pore orifice), and the resistance inside the pore, respectively^28^,^29^. Note that *R*_acc_ ≫ *R*_pore_, which makes *I*_ion_ particularly sensitive to motions of analytes at the channel entrance^22^.

When loading carboxylated polystyrene (PS) beads (diameter 780 nm) dispersed in buffer into the *cis* compartment, we observed resistive pulses in the ionic current versus time (*I*_ion_ − *t*) curves under *V*_b_ = 0.2 V signifying electrophoretic translocation of the negatively-charged PS particles through a pore. We were able to detect the resistive pulses even when sinusoidal voltage of *V*_AC_ *sin*(*ωt* + *θ*) with *V*_AC_ = 0.1 V or 1.0 V and *ω* in a range from 2 MHz to 10 MHz was added to the embedded electrodes in a configuration of a quadrupole trap^30^,^31^ by removing high-frequency components with 100 kHz Bessel low-pass filter (Fig. 1e). There were no distinct effects of the AC fields on the open pore ion transport except a slight increase in the noise level.

We explored the possible contributions of the dielectrophoretic traps on the polymeric particle translocation dynamics by inspecting the line profiles of individual resistive pulses. The height *I*_p_ and width *t*_d_ of the ionic signatures (Fig. 1f) are useful parameters describing the magnitude of ion blockage and the time-of-flight of a particle to pass through the micropore, respectively. The scatter plots revealed positive correlation between the PS bead residence time and the resistive pulse height irrespective of the AC voltage imposed (Fig. 2a–c). This is interpreted as a characteristic feature representing the off-axis effects on the ionic current blockade by spherical objects in low-aspect-ratio pore sensors^22^: the ion transport is impeded more (less) effectively when the particles pass through a position closer to (further from) the side wall, due to the non-uniformity in the electric field distributions induced by broken symmetry in the particle-in-channel system in case of off-center translocations^32^,^33^, whereat the hydrodynamic drag induced by the electroosmotic flow in direction opposite to the electrophoresis is stronger (weaker), thus leading to the correlation of higher (lower) *I*_p_ when longer (shorter) *t*_d_^22^. We also note that a size variation in the polymeric particles would also affect the ionic spike signals contributing to expand the distributions of *I*_p_ and *t*_d_. The actual influence in the present experiment is, however, anticipated to be negligibly small considering the relatively small nominal size variation in the PS particle employed (less than 5% in diameter) compared to the relative strong dependence of the current signatures on the off-axis effects^22^.

Investigating the fine structures of the resistive spike waveforms in more detail, it was found that the trace shapes differ markedly in every event in the pre-translocation regime wherein the *I*_ion_ sharply drops by the amount *I*_p_ upon single-particles entering into the micropore (Fig. 2d; see also Fig. S3). The varying *I*_ion_−*t* curve profiles are interpreted as denoting wide variations in the particle capture dynamics at the orifice upon drawn electrophoretically into the channel considering the semi-remote sensing capability of the low-aspect-ratio pore sensors^22^. The ionic profiles can be categorized into three groups according to the waveforms (Fig. 2e) characterized as sharp (orange), blunt (cyan), and single-stepped spikes (purple). The former two can be assigned to the polymeric beads passing through the center (orange) and out-of-center positions (cyan) in the pore considering the aforementioned off-axis effects^32^,^33^. On the other hand, the step signals (purple) imply temporal trapping of the PS particles on the nanoelectrodes thereby partially occluding the micropore to cause the *I*_ion_ drops in prior to the eventual translocation (Fig. 2f).

In fact, each of the distinctive translocation processes was found to involve different degrees of ionic current blockage. This can be visualized in the overplots of selected ionic signals with respect to the pulse height (Fig. 3a–c): Whereas the current spikes of *I*_p_ in a range from 0.9 nA to 1.1 nA demonstrated the three types of waveforms (Fig. 3a; see also Fig. S4), the larger and smaller spikes were found mostly the stepped (Fig. 3b) and the weak pulses, respectively (Fig. 3c), the results of which corroborate the pronounced off-axis effects on *I*_ion_ in low-aspect-ratio pores.

We evaluated the feasibility of dielectrophoresis for particle motion control by analyzing the AC dependence in the relative occurrence *f* of the characteristic events, i.e. transit through the pore center, near the side wall, and that involving temporal-block at the nanoelectrodes. While no conspicuous change in *f* was observed over the *ω* range examined under a low AC voltage condition (*V*_AC_ = 0.1 V), high-field traps at *V*_AC_ = 1.0 V effectively modulated the modes of translocation (Fig. 3d,e). Specifically, the probability increased for the off-axis trafficking as well as the temporal trap on the nanoelectrodes upon increasing the transverse voltage frequency from zero to 2 MHz, which indicates prominent AC field effects to attract the PS particles toward the electrode surface. Meanwhile, the polymer beads in turn tended to flow through the pore center under higher *ω*.

The nontrivial dependence of the particle electrophoresis on the trap voltage frequency reflects the interplay of alternating electric fields and polarizable PS particles. Theoretically, the permittivity of particles under an AC field ε^*^ is composed by real and imaginary parts (See Theoretical Derivation in Supplementary Material)^23^:


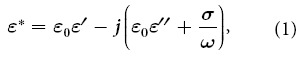


where ***ε*****″** characterizes the response of bound charges to the AC field and ***σ*** is the conductivity of the target which denotes the response of free charges. The dielectrophoretic force *F* on the polymeric bead under the effective electric field *E* is then evaluated as


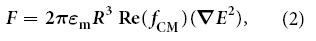


where *ε*_0_ and *ε*_m_ are the permittivity of vacuum and the solution while *ε*_p_, *σ*_p_, and *R* denote the permittivity, the conductivity and the radius of the particle, respectively. As depicted by the equation, the real part of Clausius–Mossotti factor 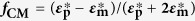 determines the sign of *F*. Our experimental observations of the force direction reverse indicate that there existed a sign change of Re(*f*_CM_) when the AC voltage frequency became larger than a critical value (about 5 MHz). This sign change suggests that either the response of bound charges or that of free charges within the PS particles to the AC field varied significantly with increasing AC frequency. By comparing the magnitudes of ***ε***′ and ***ε***″ of water and PS particles, we conclude that the movement of free charges dominated the force change while the contribution of bound charges was negligible small. Here we give a brief quantitative analysis (see detailed discussions in the Supplementary Material 2. Theoretical modelling of dielectrophoresis). The complex permittivity of water and PS particles of *ε’*_m_ = 78, *σ*_*m*_ = 2 × 10^−4^ S/m, *ε’*_*p*_ = 2.5 and *σ*_*p*_ = 20 × 10^−3^ S/m yields positive Re(*f*_CM_) at *ω* = 2 MHz^34^, which predicts the dielectrophoretic forces pointing along low-to-high electrical field directions, i.e. the particle would be pushed from the center of device to the transverse electrode region (Here, the value of *σ*_*p*_ is assumed to fit the experimental finding of the cross-over from positive-to-negative dielectrophoresis between 2 MHz and 5 MHz, which is in agreement with the previous reports within a factor of two^31^,^35^). Increasing the frequency to 5 MHz, on the other hand, changes the sign of Re(*f*_CM_) indicating inversion of the trap field acting to collect the beads to the electrode gap center (Fig. 3f). Here, the underlying mechanism is that the field-driven motions of free charges within the particles (*σ*_p_) determine the permittivity at a low frequency (*ω → *0) thereby inducing positive dielectrophoresis. As the frequency becomes higher (*ω → *∞), on the other hand, the difference between the perturbation of bound charges in the bead (*ε*_p_) and that in the water (*ε*_m_) tends to be the dominant factor that leads to negative dielectrophoresis due to phase-lag effects^23^. As the levels of *ω* are comparable to the charge relaxation frequency of 19 MHz in the electrolyte solution^36^, screening by ions is anticipated to be ineffective leading the applied transverse voltage to drop largely not at the electric double layers formed on the electrode surface but across the buffer in the pore^37^,^38^ and therefore making the AC traps efficacious to affect the particle translocation dynamics. This is the physical origin responsible for the *ω*-dependent translocation pathways that serves to validate the efficacy of the dielectrophoretic approach for controlling the particle dynamics in pore channels.

The efficiency of the AC-field to regulate the particle translocation motion is demonstrated in the change in variations of the resistive pulse waveforms. *I*_p_ and *t*_d_ histograms reveal a single-peak feature at positions *I*_c_ and *t*_c_, respectively, as defined by Gaussian fitting (Fig. S5a,b). Plots of *I*_c_ and *t*_c_ with respect to *ω* clearly depict the low (high) resistive heights with short (long) translocation duration for the particle translocating through the center (off-axis) regions of the pore at 2 MHz (5 MHz) (Fig. S5c,d). At the same time, the results illustrate the narrower distributions in *I*_p_ and *t*_d_ when subjecting the transverse electrostatic stimulus whereby corroborating the capability of the dielectrophoretic traps to finely manipulate the transverse dynamics of particles passing through a channel.

The transverse field played a role on the particle kinetics in the post-translocation regime as well. In sharp contrast to the random feature of the *I*_ion_ spike waveforms in the capture stage, the traces were well-reproduced after the transits irrespective of *ω*, the invariance of which suggests regulated migration directions of particles by virtue of the two-dimensional spatial constraint in the pore upon trafficking^22^. Interestingly, the time-of-flight *t*_es_ of the PS beads to leave the micropore, estimated from the *I*_ion_ spikes as in Fig. 4a, became shorter with increasing the transverse voltage frequency (Fig. 4b,c). The results suggest reduced hydrodynamic dragging in the AC-biased pore: Whereas the native negative surface charges on the SiN and SiO_2_ pore wall induces a cationic flow in direction opposite to the electrophoretic motions of the negatively-charged PS particles under no transverse field^39^, the high-frequency alternating voltage virtually cancel the net counterion motions thereby weakening the electroosmotic flow (EOF) to retard the particle translocation dynamics^40^. It is also noted that in addition to DC-driven hydrodynamics, AC-derived EOF velocity^41^ would be diminished rapidly with increasing *ω* by the fact that the inefficient screening by ions under the high-frequency conditions leads to little potential drop at the electric double layer^37^,^38^, and hence less counterions to generate the steady water flow.

Besides tailoring the nanoscale particle trajectories, the AC trap also improved the analyte capture efficiency. The capture rate *f*_cap_ is obtained by dividing the number of the resistive pulses by the measurement time. The normalized *f*_cap_ revealed AC-assisted particle enter into the pore sensor (Fig. 4d). This suggests a significant role of focused field gradient into the pore under the dielectrophoretic voltage^42^. In addition, the facilitated particle capture is presumably due in part to the suppression of the electroosmosis that hinders the electrophoretic entry of negatively-charged particles.

While powerful at *ω* ≤ 5 MHz, the dielectrophoresis turns out to be ineffective upon increasing the voltage frequency to 10 MHz as evidenced consistently by the declined *f*_cap_ along with the broadened distributions in *I*_p_ and *t*_d_ to levels comparable to those under no transverse field (Fig. S5). The high-*ω* characteristics is ascribable to a power loss derived from an impedance mismatch in the electrode-liquid-electrode system. Conversely, it is also speculated that the low Re(*f*_CM_) at *V*_AC_ = 1.0 V imposed excessively strong centripetal force inside a pore channel that would eventually led to irregular translocation dynamics of the particles via significant effects of inertia.

The present results demonstrate the utility of dielectrophoretic traps to regulate the capture-to-translocation process of analytes using the electrode-embedded pore structures. It is anticipated that the transverse field effects can be further strengthened by using a low salt concentration solution for inefficient screening that provides more extensive electric field distributions inside pore channels. Although such conditions would deteriorate the signal-to-noise ratio in resistive pulse sensing^3^, it can be incorporated to the transverse electron tunnelling approach^43^,^44^,^45^ without any compensation as less ions would impose no influence on the single-molecule signatures and rather advantageous in viewpoints of decreased current noise in the insulating media. In this approach, electron transport through each nucleotide is measured by using two electrodes embedded in a nanopore^46^. The tunneling current is, however, extremely sensitive to the distance between the electrode surface and the molecule, giving wide distributions in the single-molecule conductance^44^. The present method is expected to be used to diminish the variation in the tunneling current by controlling the molecular conformations residing in the electrode gap through the dielectrophoretic mechanism. The four-electrode structure can be directly used as both tunneling current sensing electrodes and also for the transverse molecular dynamics control by downscaling the dimension to fit the size of nucleotides since our results already suggest that the AC component can be removed by low-pass filtering to extract the molecular signatures.

## Methods

### Fabrication of trap electrode-embedded micropores

We fabricated four-electrode-embedded micropores that consist of a micrometer-scale pore in a SiN membrane and two pairs of Pt microelectrodes facing orthogonal to each other (Fig. S2). We formed a 50 nm thick SiN membrane by etching 0.5 mm thick Si from the back of a SiN/Si wafer in KOH solution at 90 degrees Celsius. Micro-leads were then fabricated on the substrate by photo-lithography and radio-frequency magnetron sputtering processes. After that, we formed Pt electrodes of 60 nm thickness with a gap separation of 1 μm with electron-beam lithography and sputtering methods using the electrode pattern as external markers. Subsequently, we coated the substrate surface with a 25 nm thick SiO_2_ layer by chemical vapor deposition. Finally, we dry-etched the SiO_2_ and SiN through an etching mask formed with ZEP520A-7 resist material by the electron beam drawing to sculpt a hole of diameter 1.4 μm. Here, we used two-step etching to expose only the nanoscale tips of the transverse electrodes for suppressing any leakage current.

### Single-particle detections

In prior to the resistive pulse measurements, the electrode-embedded micropore were sealed with two polydimethylsiloxane blocks from the both sides wherein Ag/AgCl electrodes were inserted to apply electrophoretic voltage and detect ion current through the micropore. Carboxylated polystyrene (PS) beads of diameter 0.78 μm (ThermoScientific Inc.) were employed as analyte particles. In experiments, we filled one side of the pore with a dispersion solution of the PS beads in TE buffer (pH 8.0, Tris-HCl 10 mM, EDTA 1 mM) at a concentration of 0.3 pM and the other with the same buffer but with no particles added. We used a voltage source (Keithley 6487) to apply a DC voltage *V*_b_ and recorded the ion current flowing through the pore via the two Ag/AgCl electrodes at 250 kHz using Axopatch 200B (Axon Instruments) with a 100 kHz low-pass filter and a digitizer (NI PXI-5922) backed by a RAID system (NI HDD-8263). Meanwhile, we applied sinusoidal voltage of peak-to-peak amplitude *V*_AC_ and frequency *ω* to one pair of electrodes and π/2 phase-shifted counterpart to the other pair using a function generator.

### Data analysis

The resistive pulses of specific heights were extracted by using a computer program coded to find local current minima in *I*_ion_ − *t* curves below 300 pA and record 5 ms of data before and after the spikes when *I*_p_ is within a window.

## Additional Information

**How to cite this article**: Tanaka, S. *et al*. Tailoring particle translocation via dielectrophoresis in pore channels. *Sci. Rep.* **6**, 31670; doi: 10.1038/srep31670 (2016).

## Supplementary Material

Supplementary Information

## Figures and Tables

**Figure 1:**
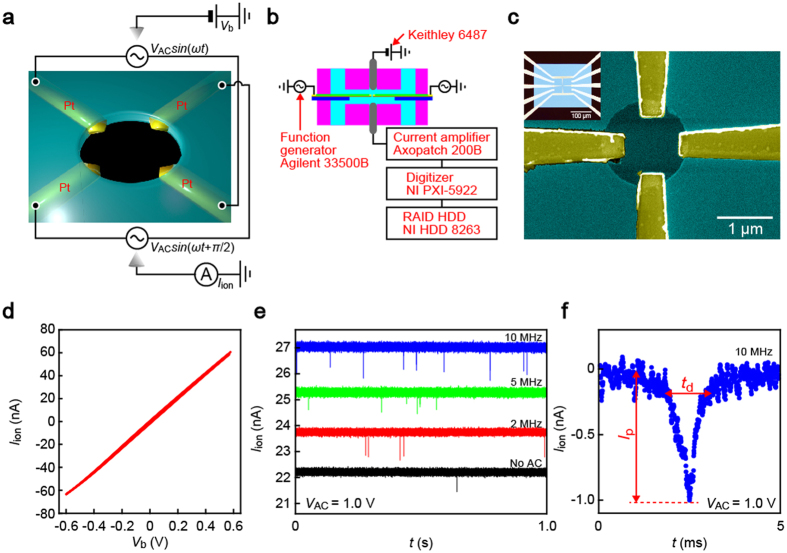
Trap electrode-embedded micropore sensor. (**a,b**) Schematic illustrations of a four-electrode-embedded micropore (**a**) and a measurement set up (**b**). Sinusoidal voltage of *V*_AC_ *sin*(*ωt* + *θ*) was applied through the embedded electrodes for dielectrophoretic trapping while the electrophoretic voltage *V*_b_ across the pore was used for resistive pulse detections of carboxylated polystyrene beads. (**c**) Scanning electron microscopy image of a micropore sensor consisting of a 1.4 μm-sized hole in a 50 nm thick SiN membrane with four Pt electrodes arranged in a cross-wise configuration. Inset is an optical view of the pore channel showing the Si_3_N_4_ membrane and the microelectrode pattern. (**d**) The cross-pore ionic current *I*_ion_ plotted as a function of *V*_b_. (**e**) Partial *I*_ion_ traces recorded at *V*_b_ = 0.2 V, V_AC_ = 1.0 V, and *ω* ranging from 2 MHz to 10 MHz. Data obtained under no transverse field is also displayed. The ionic spikes indicate single-particle translocation through the micropore. (**f** ) A close-view of a resistive pulse. *I*_p_ and *t*_d_ denote the height and width of the signal.

**Figure 2:**
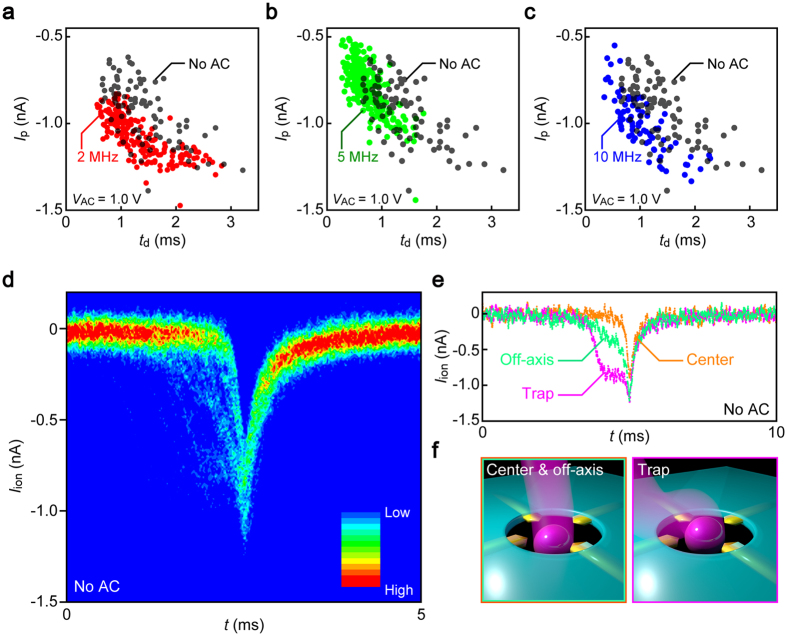
Single-particle translocation modes. (**a–c**) Scatter plots of the resistive pulse height *I*_p_ versus the width *t*_d_ measured under *V*_b_ = 0.2 V and *V*_AC_ = 1.0 V. The frequency of the AC voltage is 2 MHz (**a**), 5 MHz (**b**), and 10 MHz (**c**). Data obtained under no transverse field are also shown (black plots). (**d**) Two-dimensional histogram of 79 resistive pulses obtained at *V*_b_ = 0.2 V under no transverse field. (**e**) The three representative *I*_ion_ curves showing sharp (orange), blunt (cyan), and stepwise features (purple) in the pre-translocation regimes. (**f** ) The schematic models for the three characteristic *I*_ion_ signals.

**Figure 3:**
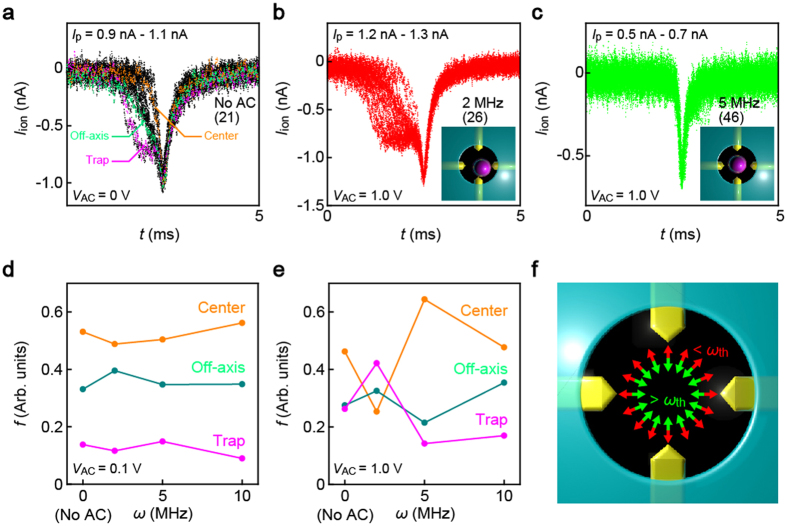
Transverse field controlled single-particle translocation. (**a–c**) Overplots of equi-*I*_p_ resistive pulses collected from the data obtained under *V*_b_ = 0.2 V and *V*_AC_ = 1.0 V with different transverse AC voltage frequency and *I*_ion_ windows: No transverse field with a 0.9 nA–1.1 nA window (**a**); *ω* = 2 MHz with a 1.2 nA–1.3 nA window; and *ω* = 5 MHz with a 0.5 nA–0.7 nA window. The number in the parentheses denote the number of signals plotted superimposed. Insets are the images illustrating translocation passages involved in the data. (**d,e**) The relative occurrence of the three distinct translocation modes, i.e. particle transits through the pore center (orange), off-axis positions (green), and those accompanying temporal traps at the embedded electrodes (purple). (**f**) Schematic explanations of the transverse field-dependent dielectrophoretic forces pointing toward the electrodes (red) and the pore center (green) at voltage frequencies lower and higher than the threshold *ω*_th_, respectively.

**Figure 4:**
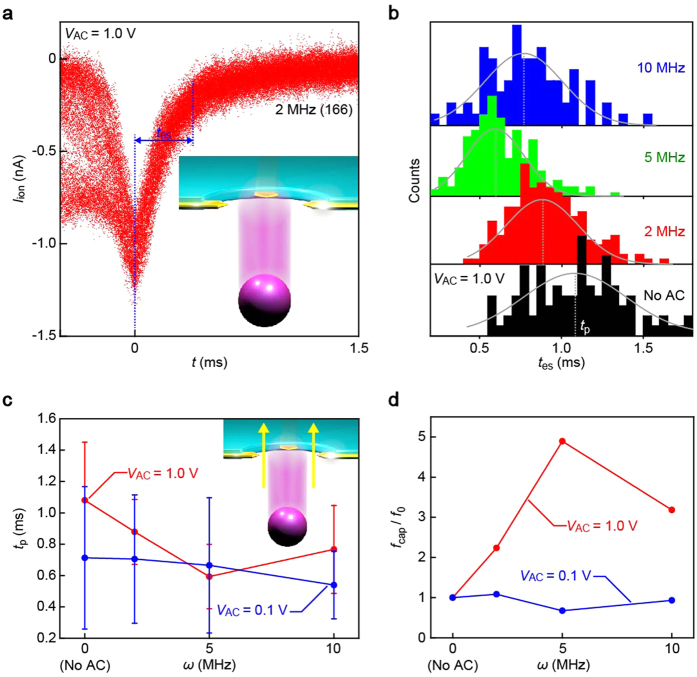
Field-modulated particle capture and post-translocation dynamics. (**a**) *I*_ion_–*t* overplots of 166 signals recorded at *V*_b_ = 0.2 V, *V*_AC_ = 1.0 V, and *ω* = 2 MHz. The time required for particles to escape from the micropore (defined as the duration for I_ion_ to decrease from *I*_p_ to 0.3*I*_p_) *t*_es_ was extracted from each pulse. (**b**) AC voltage frequency dependent *t*_es_ distributions. Solid lines are Gaussian fitting to the histograms defining peak positions *t*_p_ used to evaluate the trap-field dependence of the post-translocation particle kinetics. (**c**) *t*_p_ plotted against the AC voltage frequency *ω* suggesting shorter escape time, and hence faster speed, of particles during escape from the micropore. Error bars are the full-width at half-maxima of the Gaussian distributions fitted in (**b**). *t*_p_ increased at 10 MHz presumably due to the impedance mismatch causing partial reflection of the high-frequency voltage in the nanoelectrodes. (**d**) The particle capture rates *f*_cap_ normalized by *f*_cap_ under no AC field, *f*_0_. The plots show AC-assisted particle capture into the pore. The turnover at 10 MHz is attributable to the impedance mismatch.
