# Supplementary material for: Tailoring particle translocation via dielectrophoresis in pore channels
Source: Sci Rep. 2016 Aug 16;6:31670. doi: 10.1038/srep31670 (PMC4985646; doi:10.1038/srep31670)
Supplement: Supplementary Information [file srep31670-s1.pdf]

Supplementary Information for

Tailoring particle translocation via dielectrophoresis in pore channels

Shoji Tanaka[1,2], Makusu Tsutsui[1], Hu Theodore[3], He Yuhui[3], Akihide Arima[1], Tetsuro Tsuji[2], Kentaro Doi[2], Satoyuki Kawano[2], Masateru Taniguchi[1], and Tomoji Kawai[1]

[1]*The Institute of Scientific and Industrial Research, Osaka University, Ibaraki, Osaka 567-0047, Japan*

[2]*Department of Mechanical Science and Bioengineering, Graduate School of Engineering Science, Osaka University, Toyonaka, Osaka 560-8531, Japan*

[3]*School of Optical and Electronic Information, Huazhong University of Science and Technology, Luo Yu Road, Wuhan 430074, China*

The Supplementary Information includes:

1. **Supplementary Figures (Figure S1-S7)**

2. **Theoretical modelling of dielectrophoresis**

1. Supplementary figures

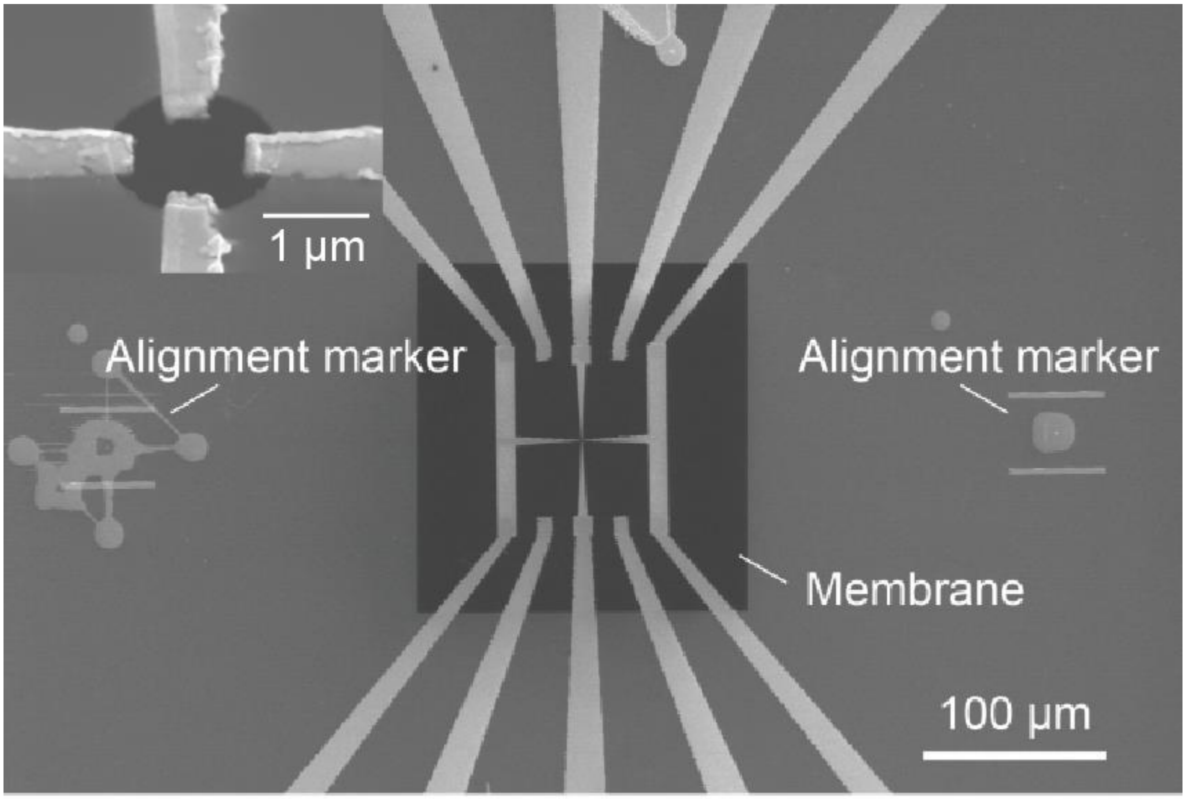

Figure S1. Scanning electron microscopy image of a quadrupole-electrode-embedded micropore. The dark square region is a 50 nm thick SiN membrane formed on a 0.5 mm thick Si wafer. The two alignment markers at the both sides were used to delineate the electrode and pore patterns with a standard electron beam lithography process. The four electrodes were connected to microleads to feed through the dielectrophoretic field inside the pore. Inset is a magnified view of the four-electrode-embedded micropore. The image was taken with the substrate inclined by 45 degrees.

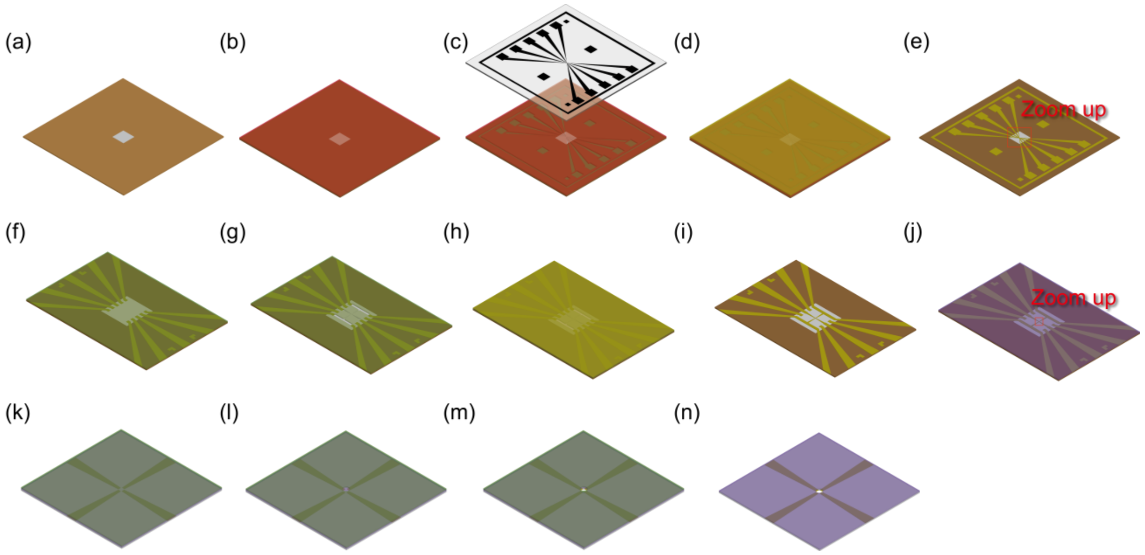

Figure S2. Fabrication processes of quadrupole-electrode-embedded micropores. (a) On a Si wafer having a 50 nm thick SiN membrane, (b) photo-resist (AZ5206E) was coated. (c) By irradiating ultraviolet light over a mask and subsequent development, microelectrode pattern was formed in the resist. (d) Then, a thin Cr/Au layer of thickness 30 nm was deposited by radio-frequency magnetron sputtering. (e) After lift-off in N'N-dimethylformamide, we obtained five pairs of microelectrodes. (f) After that, EB resist (ZEP520) was spin-coated and (g) four nanoelectrodes were rendered via electron beam lithography followed by development. (h) What followed was deposition of a Pt layer by the sputtering and (i) lift-off. As a result, we acquired quadrupole electrodes on the membrane. (j) EB resist was further coated on the substrate. (k) A pore was then EB-drawn at the center of the gap between the four electrodes (l) followed by development. (m) By using the resist layer as a mask, a micropore was sculpted by exposing the substrate

to a reactive-ion etching (etchant gas: CF_4). (n) Finally, the remnant resist was removed by immersing the sample in N’N-dimethylformamide overnight.

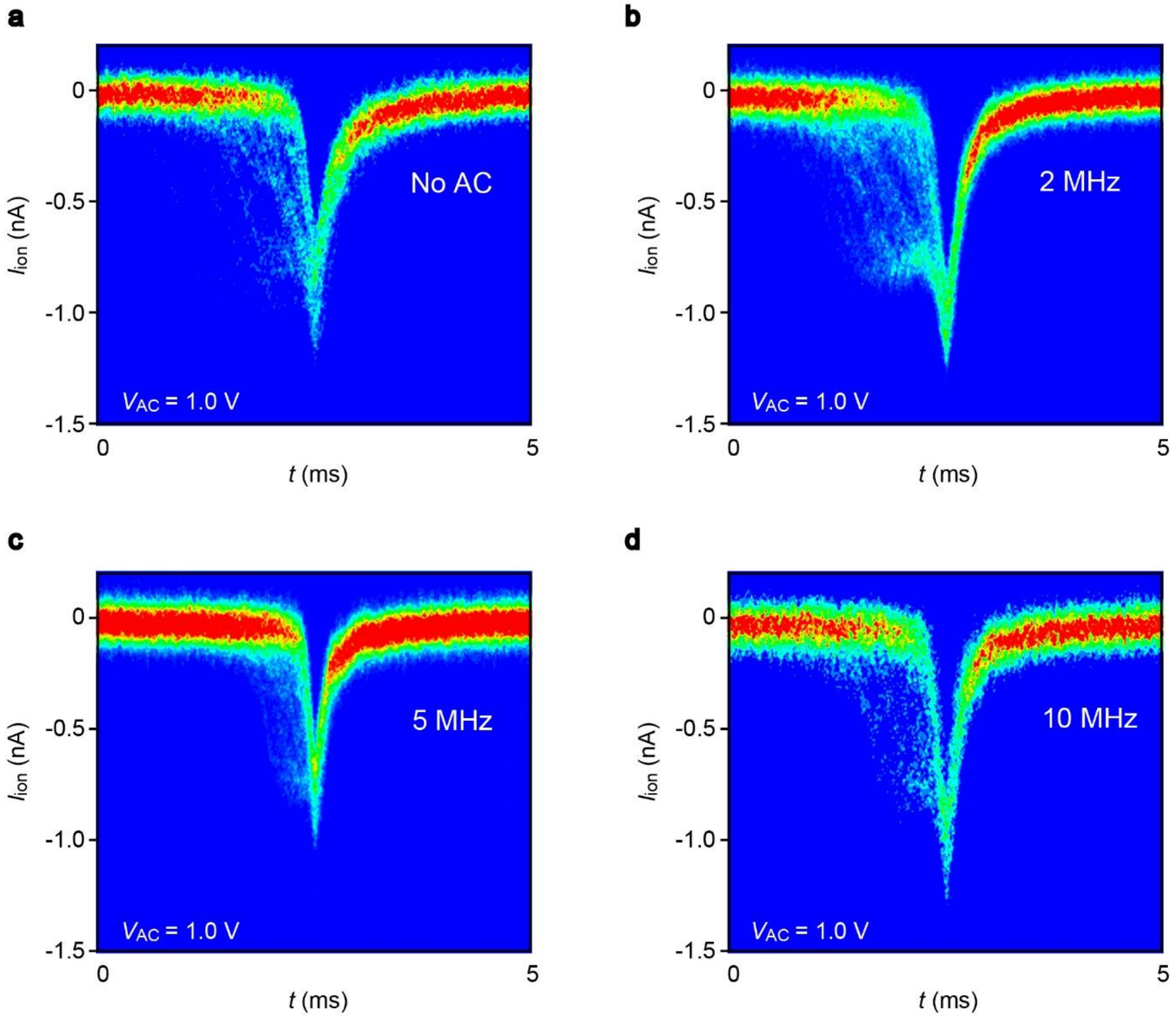

Figure S3. Two-dimensional histograms of resistive pulses measured using the quadrupole-electrode-embedded micropore at the electrophoretic voltage of 0.2 V. **a,** Results for no AC voltage added to the transverse electrodes. **b-d,** The histograms for the cases when sinusoidal trap voltage was added to the quadrupole electrodes with amplitude V_{AC} = 1.0 V and frequencies of 2 MHz (b), 5 MHz (c), and 10 MHz (d).

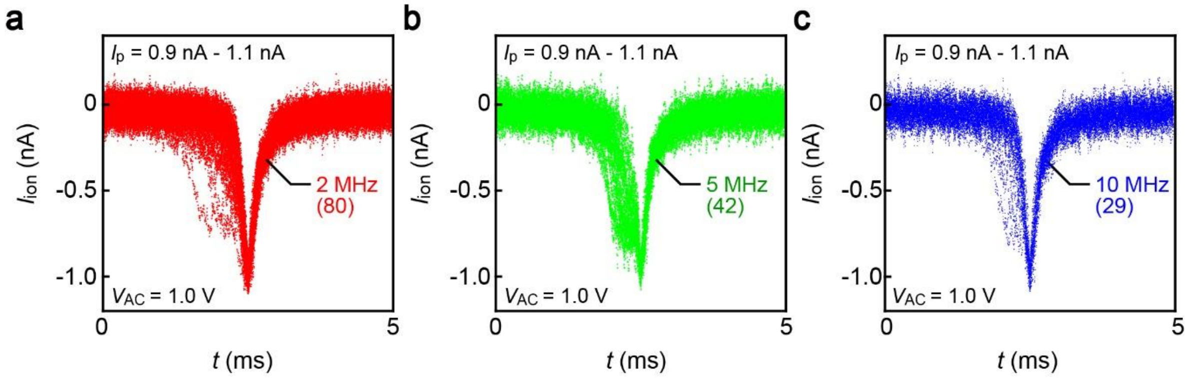

Figure S4. Resistive pulses of height I_p in a range from 0.9 nA to 1.1 nA extracted from the data obtained at the electrophoretic voltage of 0.2 V with the transverse AC voltage of amplitude V_{AC} = 1.0 V and frequencies ω = 2 MHz (a), 5 MHz (b), and 10 MHz (c). The number in the parentheses indicates the number of resistive spikes overplotted.

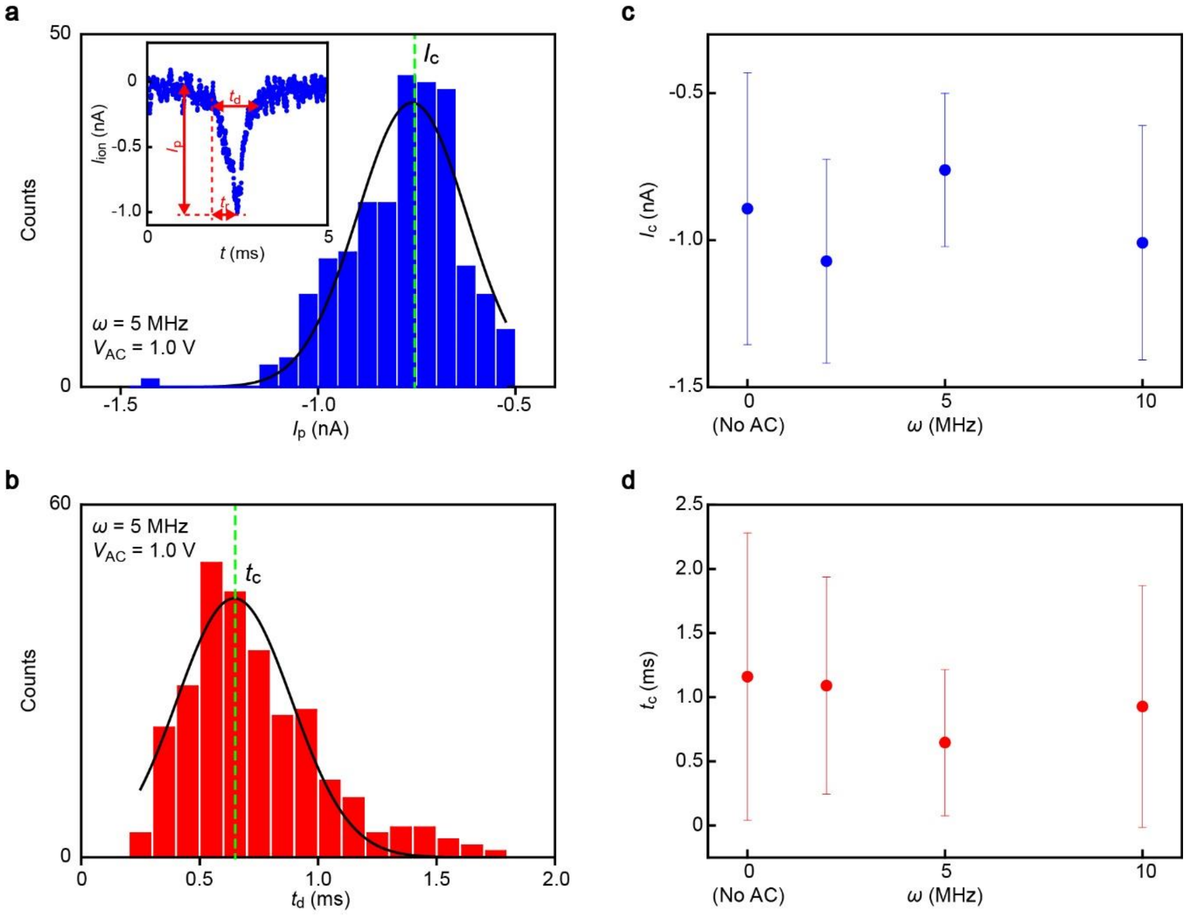

Figure S5. a-b, Distributions of the height I_p (a) and width t_d (b) obtained with the AC transverse voltage with amplitude V_{AC} = 1.0 V and frequency ω = 5 MHz applied to the quadrupole electrodes. Solid curves are Gaussian peaks fitted to the histograms. The dotted lines denote the positions I_c and t_c of the single Gaussian peak. **c-d,** I_c (c) and t_c (d) plotted against ω. Error bars indicate the full-width at half maximum of the Gaussian distribution shown in (a) and (b). Note that the variations in I_p and t_d tend to be smaller as increasing ω to 5 MHz suggesting well-regulated particle translocation motions under the AC field-induced dielectrophoresis in the pore.

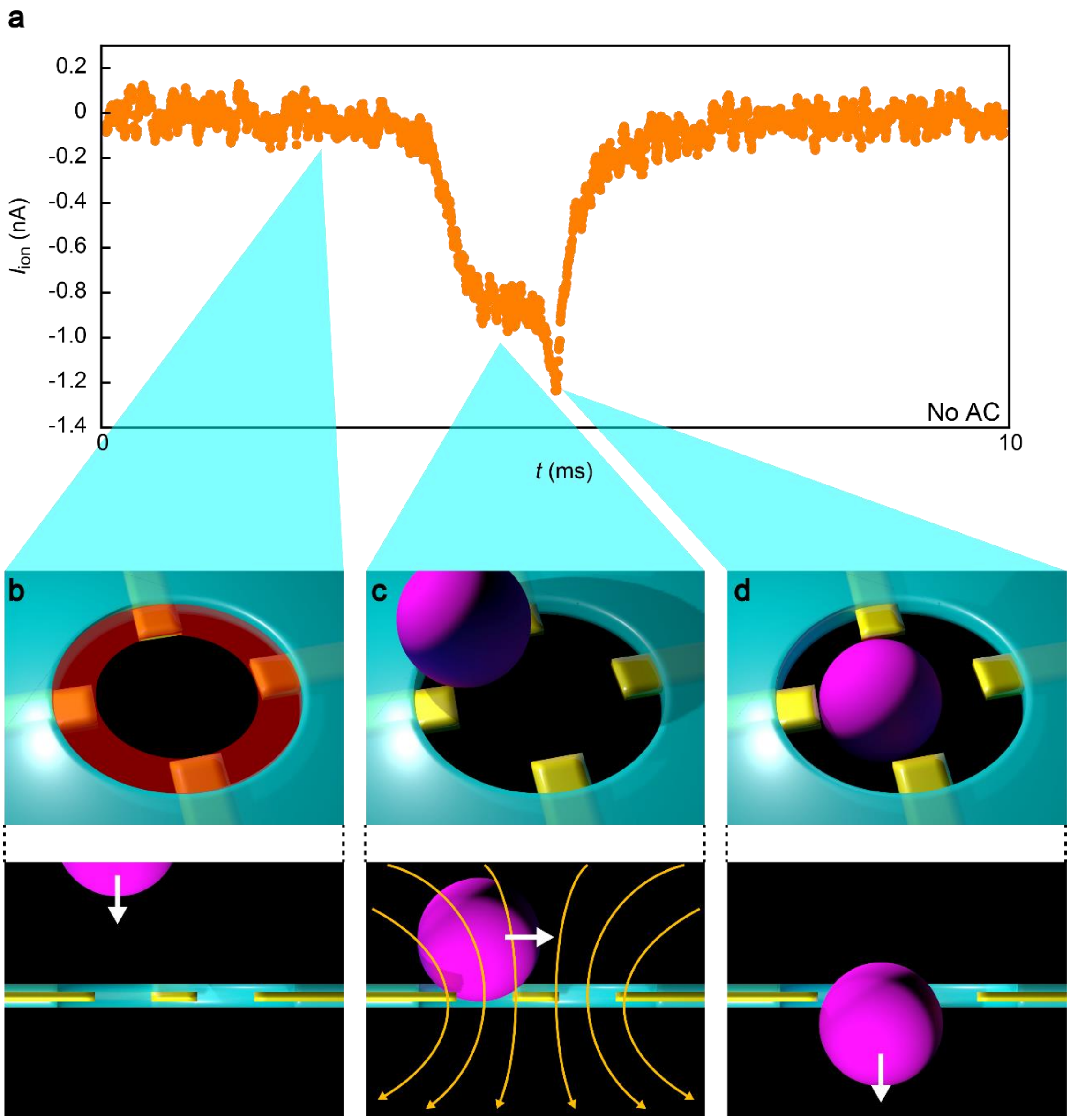

Figure S6. Single-stepped ionic spikes and an underlying particle trap mechanism. **a,** An ionic spike having a small step suggestive of temporal trapping of particles in vicinity of the micropore. **b-c,** Particles captured at the region outside the electrode gap (red-coloured area (b)) cannot pass through the pore but instead being trapped by the electrophoretic force at the pore orifice (c). Partial blocking of the ion transport by the trapped particle gives rise to the single-step feature. **d,** Meanwhile,

the electric field moves the particle toward the center region until it is allowed to translocate through the conduit.

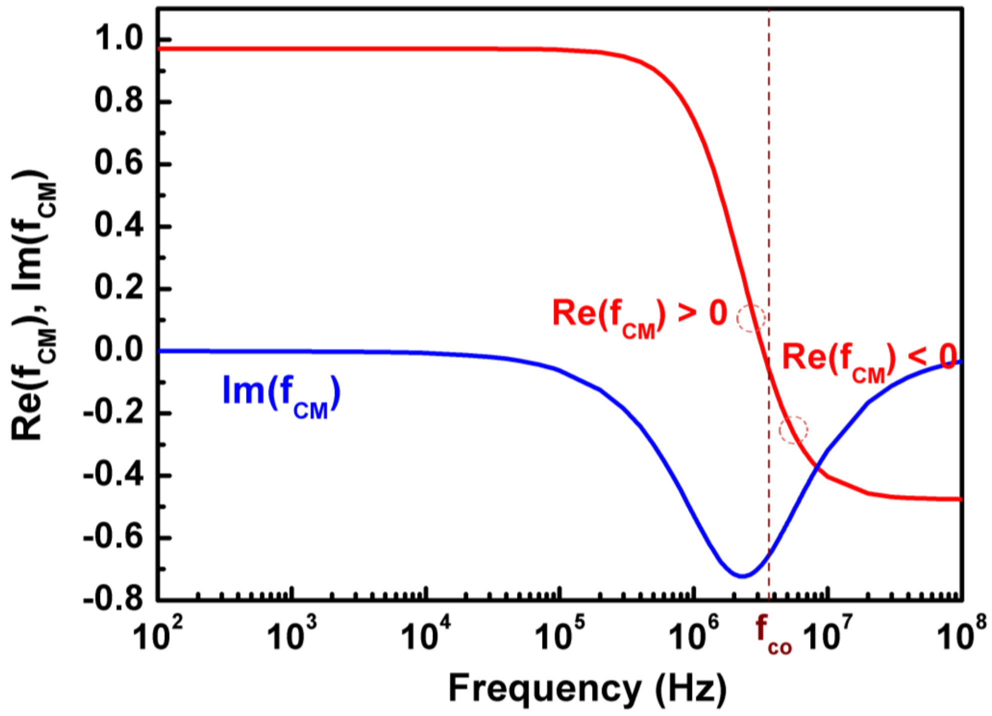

Figure S7. The real and imaginary parts of Clausius-Mossoti factor as functions of the AC transverse voltage frequency. f_{co} is the cross-over frequency indicating the frequency at which the sign of Re(f_{CM}) changes from positive to negative: For $f<f_{co}$, Re(f_{CM})>0 and the dielectrophoretic force acts toward the high electric field region (electrode), while for $f>f_{co}$, Re(f_{CM})<0 and the dielectrophoretic force acts toward the low electric field region (trap center). The two dash circles denote that Re(f_{CM})>0 at f = 2MHz, while Re(f_{CM})<0 at f = 5MHz. Here we set $\sigma_p = 20\times10^{-3}$ S/m and ε_p = 2.5 [4].

2. Theoretical modelling of dielectrophoresis

The Maxwell equation involved in our situation is written as

$$\nabla \cdot \vec{D}(\vec{r}, t) = 0$$

In our experiment, an AC voltage with frequency ω was applied to the transverse electrodes: $V(\vec{r}, t) = V(\vec{r})e^{i\omega t}$. Thus monochromatic waves were assumed:

$$\vec{D}(\vec{r}, t) = \vec{D}(\vec{r})e^{i\omega t},\ \ \vec{E}(\vec{r}, t) = \vec{E}(\vec{r})e^{i\omega t}$$

and the electric displacement was related to the electrical field via the dielectrics:

$$\vec{D}(\vec{r}) = \varepsilon\varepsilon_0\vec{E}(\vec{r})$$

Here we remind that ε was dependent on ω.

The response of free charges and that of bound charges in the dielectric medium to the AC field are quite different. The former ones can be described in term of conductivity σ, and the latter with the imaginary part of the permittivity:

$$\varepsilon = \varepsilon_0(\varepsilon' - j\varepsilon")$$

In the above $\tan^{-1}(\varepsilon"/\varepsilon')$ denotes the phase lag caused by the deterrent response of bound charges to the AC field.

According to Maxwell equation:

$$\nabla \times \vec{H} = \frac{\partial}{\partial t}\vec{D} + \sigma\vec{E} = [j\varepsilon_0\omega(\varepsilon' - j\varepsilon") + \sigma]\vec{E}(t)$$

Thus, we arrive at the following expression for the complex permittivity [1]:

$$\varepsilon^* = \varepsilon_0(\varepsilon' - j\varepsilon" - j\frac{\sigma}{\varepsilon_0\omega})$$

For water, $\varepsilon'_m \approx 78$, $\sigma_m \approx 2 \times 10^{-4}$ S/m, $\frac{\sigma_m}{\varepsilon_0\omega} = \begin{cases} 4.52 & \omega = 5\text{ MHz} \\ 11.3 & \omega = 2\text{ MHz} \end{cases}$, $\varepsilon''_m = \begin{cases} 0.0035 & \omega = 5\text{ MHz} \\ 0.0014 & \omega = 2\text{ MHz} \end{cases}$, [2]. Hence we consider the contribution by the free charges, and neglect that by the bound charges only [2]:

$$\varepsilon^*_m = \varepsilon_0\varepsilon'_m - j\frac{\sigma_m}{\omega}$$

On the other hand, for PS particles, $\sigma_p \approx 20 \times 10^{-3}$ S/m, $\frac{\sigma_p}{\varepsilon_0\omega} = \begin{cases} 451 & \omega = 5\text{ MHz} \\ 1129 & \omega = 2\text{ MHz} \end{cases}$, while $\varepsilon'_p = 2.5$ [3], and $\varepsilon'_p > \varepsilon''_p$. Therefore, the bound charge response within the PS particles is negligible compared to the free charge counterpart:

$$\varepsilon^*_p = \varepsilon_0\varepsilon'_p - j\frac{\sigma_p}{\omega}$$

So, the Clausius-Mossotti factor becomes

$$\mathrm{Re}(f_{\mathrm{CM}}) = \frac{\left(\varepsilon'_p - \varepsilon'_m\right)\left(\varepsilon'_p + 2\varepsilon'_m\right) + \dfrac{(\sigma_p - \sigma_m)(\sigma_p + 2\sigma_m)}{\varepsilon_0^2\omega^2}}{(\varepsilon'_p + 2\varepsilon'_m)^2 + \dfrac{(\sigma_p + 2\sigma_m)^2}{\varepsilon_0^2\omega^2}}$$

Our calculation results are demonstrated in Fig.S7.

where ω_{pl} is the plasma frequency.

[1] S. Ramo, J.R. Whinnery, and T. Van Duzer, Fields and Waves in Communication Electronics, 3rd ed., (John Wiley and Sons, New York, 1994). ISBN 0-471-58551-3

[2] U. Kaatze, Complex Permittivity of Water as a Function of Frequency and Temperature, J. Chem. Eng. Data 1909, 34, 371-374.

[3] B. Yafouz, N. A. Kadri and F. Ibrahim, Dielectrophoretic manipulation and separation of microparticles using microarray dot electrodes. Sensors 14, 6356-6369 (2014).

[4] N. Green and H. Morgan, Dielectophoresis of submicrometer latex spheres. 1. Experimental results, J. Phys. Chem. B 103, 41-50 (1999).
